# Design of 3D Hybrid Plant Extract/Marine and Bovine Collagen Matrixes as Potential Dermal Scaffolds for Skin Wound Healing

**DOI:** 10.1155/2022/8788061

**Published:** 2022-06-30

**Authors:** Aida Lahmar, Maroua Rjab, Fairouz Sioud, Mouna Selmi, Abir Salek, Soumaya Kilani-Jaziri, Leila Chekir Ghedira

**Affiliations:** ^1^Research Unit for Bioactive Natural Products and Biotechnology UR17ES49, Faculty of Dental Medicine of Monastir, University of Monastir, Avicenne Street, Monastir 5000, Tunisia; ^2^Department of Pharmaceutical Sciences A, Faculty of Pharmacy of Monastir, University of Monastir, Avicenne Street, Monastir 5019, Tunisia

## Abstract

Tissue engineering involves the use of smart biomimetic hybrid matrices to reinforce the cellular interaction with the matrix and restore native properties after regeneration. In this study, we highlight the potential of 3D collagen sponges soaked with bioactive extract, to enhance the wound healing process *in vivo*. Acid-soluble collagen from two sources, marine and bovine, were extracted and characterized physiochemically using Fourier transform infrared spectroscopy (FTIR) and SDS-PAGE. Our results confirmed that the extracted collagens were mainly composed of collagen type I with slight molecular structure differences. Both collagens present two different *α* chains (*α*1 and *α*2) and one *β* chain. Highly interconnected 3D scaffolds from collagen from the skin are designed and added by the widely known healing plants *Pistacia lentiscus* and *Calendula officinalis*. The resulting 3D collagen matrices possess fine biocompatibility with skin cells, Hacat (keratinocytes), and 3T3-L1 (fibroblasts) cells. To evaluate the potential wound healing effect, a collagen sponge soaked with the bioactive extract was tested on BALB/*c* mice. Our findings confirmed that sponges significantly improve animal re-epithelialization by increasing wound closure. Consequently, spongy collagen scaffolds loaded with *Pistacia lentiscus* and *Calendula officinalis* could be used as potential wound dressing material.

## 1. Introduction

For many years, patients with extensive deep burns have been cured with cultured epidermal sheets in burn centers. Burns are the most devastating form of trauma that has afflicted humankind since antiquity and their short- and long-term consequences leave severe sequelae in the patients concerned [1]. The second half of the 20th century saw the intensive development of regenerative medicine, burn therapy, and pharmacotherapy, involving the use of tissue scaffolds for the formation of new viable tissue and thereby reducing the reliance on donor tissue and organs [2]. However, immunogenicity, postoperative infections, and restriction of donor sites limit the use of conventional skin substitutes [3].

Therefore, natural and synthetic fibers are a good option for tissue repair, and fibrous scaffolds are mechanically stable and biologically capable of functioning at the implant site [4]. Scientists developed some smart biomimetic hybrid matrices to reinforce the cellular interaction with the matrix and restore native properties after regeneration [5]. These products may not be the perfect replacement for natural skin; however, they may meet the need of skin graft need by providing immediate protection to the wound and improved tissue regeneration after injury [6]. Collagen-based biomaterials involve the extraction, purification, and polymerization of collagen to form a functional scaffold. Due to tissue-mimicking characteristics, it provides cell-cell and cell-matrix interactions with the fibroblast by the formation of new granulation tissue and epithelium [7].

Collagen I, which is the main component of the extracellular matrix, plays a crucial role in maintaining tissue remodeling, biological integrity, and structural mechanics [8]. Such prevalent functions make collagen I a promising material in skin tissue engineering [9].

The source of collagen is purified from animal sources or as an integral component of a more complex extracellular matrix, and its treatment before use is an important variable in the design of tissue-engineered devices. Wound dressing with a collagen sponge can be a requirement, such as increased gas permeation and protection of the wound from infection and dehydration [10]. The healing of an infected wound needs systemic administration of antimicrobial drugs. Some studies have overcome successfully wound infection by topical application of antibiotics with collagen to control infection [11, 12]. The inappropriate use of antibiotics in human medicine therapy is the main cause of the development of bacterial resistance to antibiotics [13]. Plant-based active compounds seem to be a good alternative.

The traditional importance and pharmacologically effective plants, *Pistacia lentiscus* and *Calendula officinalis,* are commonly known and widely used as a traditional medicine for wound healing to treat various skin diseases [14, 15]. *Pistacia lentiscus* is widely available in Asia, the Mediterranean region of Europe, and Africa. Over the decades, the villagers used crude leaf extracts as a traditional medicine for wound healing that have antioxidant and antibacterial properties to treat various skin diseases and wound healing [16]. In the Mediterranean region, the different parts of *P*. *lentiscus* (roots, leaves, fruits, and mastic) exhibited several medicinal uses, which have been reported in several traditional pharmacopoeias. In particular, fruit oil is used for respiratory allergies, to treat ulcers, sore throats, and applied for wounds and burns [17]. Marigold or *Calendula officinalis* was already used in medicine and cosmetics by the ancient Indian, Arab, and Greek civilizations. The flowers and roots of this plant are traditionally used to treat skin inflammation, eczema, wounds, and varicose ulcers [18]. Several studies revealed that marigold is a promising plant with an effective therapeutic potential against skin diseases [19–21]. Collagen scaffolds are mechanically stable and able to function biologically at the implant site. Various collagen-based materials have been used, including particles, fibers, films, sponges, and hydrogels, which can act as cell-supporting scaffolds or cell encapsulating matrices [22]. However, loading these scaffolds with known healing plants can perfectly, replace skin grafts by providing immediate protection to the wound and improving the quality of tissue regeneration and by providing would area with the antioxidant and proliferative properties coming from plant extracts [23]. The combination between collagen biomaterials and vegetable sources is poorly studied despite they present a promising approach.

The study aims to design for the first time a 3D hybrid collagen matrix impregnated with bioactive extracts of *Pistacia lentiscus* and *Calendula officinalis* for wound dressing and in vivo wound healing, to produce a cultured dermal substitute for skin defects and burn wounds.

## 2. Material and Methods

### 2.1. Materials

Bovine face pieces were purchased from a local tannery. *Scyliorhinus canicula* fish skin waste was collected from the fish market, Monastir, Tunisia, and stored at −20°C until used. Sodium hydroxide (NaOH), sodium chloride (NaCl), and butanol (99%) were purchased from Loba Chemie, and acetic acid was purchased from Merck India Ltd. Acrylamide, bis-acrylamide, Tris-HCl, SDS (sodium dodecyl sulfate), ammonium persulfate, TEMED (tetraacetylethylenediamine), glycerol, *β*-mercaptoethanol, bromophenol blue, and Coomassie R-250 were purchased from Sigma-Aldrich.

### 2.2. Collagen Extraction

**Figure 1:**
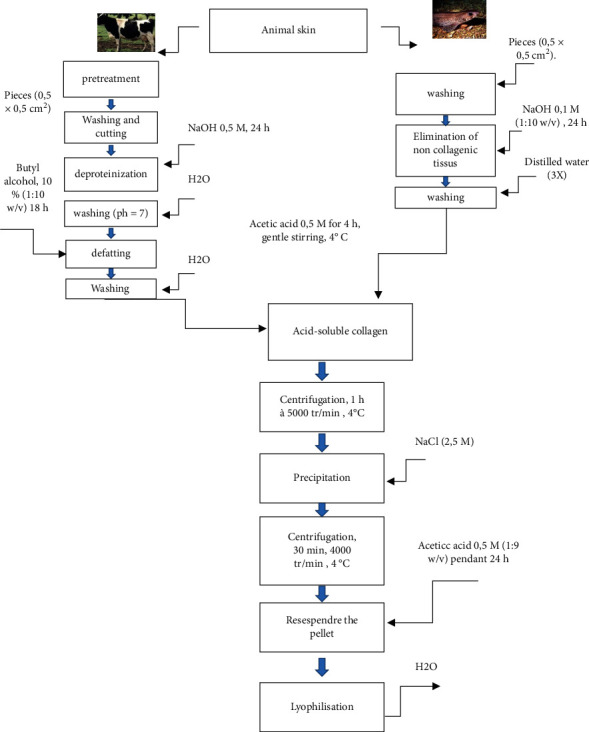
Flow sheet of the procedure used to extract collagen from two origins: Fish and bovine skins.

Marine and bovine collagen were extracted by solubilization in an acid solution, according to Matmaroh et al. [24] with some modifications. Bovine hide off-cuttings were dehaired and bleached in 0.5 M NaOH for 24 h prior to size reduction. Then, both bovine and fish fresh skin pretreated with 0.5 M NaOH for 24 h (to eliminate proteins) was washed with distilled water and then cut into small pieces (0.5 × 0.5 cm^2^) (Figure 1).

Approximately, 5 g of each skin was weighed and mixed with 0.1 M NaOH (1 : 10 w/v), for 6 h at 4°C under agitation to remove noncollagenous tissue (deproteinisation). The skin was washed with distilled water until a neutral pH was reached and then treated with 10% butyl alcohol (1 : 10 w/v) for 18 h to remove fat (this step is only for bovine skin). The samples of both origins were washed 3 times with distilled water and suspended in 0.5 M acetic acid for 48 h under gentle agitation at 4°C. The resulting solutions were then centrifuged at 5000 rpm for 1 h at 4°C and finally treated with NaCl (2.5 M) to precipitate the solubilized collagen. The precipitates were centrifugated at 4000 rpm for 30 min, and the pellets were dissolved in 0.5 M acetic acid (1 : 9 w/v) for 24 h before freeze-drying.

### 2.3. Preparation of Hydroethanolic Plant Extracts

The aerial parts of the *Pistacia lentiscus* and *Calendula officinalis* plants (leaves and flowers), freshly harvested, were dried in a dry and airy place away from light. A total of 100 g of dried powder from the aerial part of the plants were macerated with a mixture of 80% ethanol and 20% water (1L) for 72 h days at room temperature. Then, the suspension was filtered using Whatman paper No. 01 and then the filtrate was concentrated by a rotary evaporator under reduced pressure at 40°C, to remove the ethanol. Three extracts were prepared: *P. lentiscus* leaves (PL), *C. officinalis* leaves (FL), and flowers (FR). The evaporated filtrate was poured into aluminum trays and stored at −20°C for 24 h, and then freeze-dried to remove water.

### 2.4. Characterization of Collagen

- Fourier transform infrared spectra (FTIR)
- FTIR analysis was carried out to characterize the collagen solutions. The spectral measurements were measured at a resolution of 4 cm^−1^ in the frequency range of 4000–500 cm^−1^ using Carry FTIR 630, Agilent.
- SDS-PAGE
- Electrophoresis (SDS-PAGE) was performed using Laemmli (1970) method with modification [25], using a discontinuous Tris-HCl/glycine buffer system, 7% separating gel, and 4% stacking gel. A collagen solution of 2 mg/ml in sample buffer was prepared under both reducing and nonreducing conditions by the addition or exclusion of 2-*β* mercaptoethanol (SRL, Mumbai). High molecular weight marker (protein molecular weight marker; MW range 10–315 KDa; Genetix, India) and calf skin type I collagen (Fluka, Sigma-Aldrich Chemie Gmbh, Buchs, USA) were run along with samples. Electrophoresis was performed using the mini dual vertical electrophoresis unit.

### 2.5. Assessment of *In Vitro* Biocompatibility of Collagen Solutions and Plant Extracts

3T3-L1 (fibroblasts) and HaCat (keratinocytes) cells were in a 96-well plate and allowed to attach overnight. Then, cells were incubated in the presence of different concentrations of collagen and solutions and different plant extracts (6.25, 2.5, 25, 50, and100 *μ*g/mL) for 24 h, 48 h, and 72 h. After incubation, 40 *μ*L MTT (5 mg/mL) was added and incubated for 2–4 h at 37°C to observe the purple colour and the absorbance was determined at 570 nm. This optical density corresponds to the absorbance of the living cells present in each well [26].

### 2.6. Fabrication of a 3D Collagen Sponge Incorporated with Plant Extracts

Highly interconnected scaffolds from skin collagen were designed: viscous solution of collagen 20% (W/V) is added by plant extract (1 mg/mL). Then, the solution was mixed in a blender for 15 min to obtain a homogeneous collagen solution and neutralized by 4 M NaOH. The homogeneous solution was dialyzed in deionized water and lyophilized, sterilized by Co 60 and stored at 4 C for utilization [27].

Eight types of sponges were prepared:

1. Marine collagen (CP)
2. Marine collagen + hydroethanol extract of *Pistacia* leaves (CP + PL)
3. Marine collagen + hydroethanol extract of *Calendula* leaves (CP + FL)
4. Marine collagen + hydroethanol extract of *Calendula* flowers (CP + FS)
5. Bovine collagen alone (CB)
6. Bovine collagen + hydroethanolic extract of *Pistacia* leaves (CB + PL)
7. Bovine collagen + hydroethanolic extract of *Pistacia* leaves (CB + FL)
8. Bovine collagen + hydro-ethanolic extract of *Calendula* flowers (CB + FS).

### 2.7. *In Vivo* Study

#### 2.7.1. Excision Wound Model

Mice (*n* = 8) were anaesthetized by intraperitoneal administration. The skin was disinfected with alcohol, and an excisional wound was made using a 6 mm diameter punch. The wounds of the control group were protected by a conventional gaze. Sponges were changed every 2 days for 15 days to move unwanted cells from the wound bed. It is worthwhile to study the effect of such temporary coverings on the reduction of wound healing period compared to conventional gauze. The wounds of the treated groups of mice were covered with different collagen extract sponges [28].

During the healing period, the wounds were regularly measured and photographed (every 2 days). The rate of shrinking of the epithelium of wounds was evaluated to have statistical significance between the groups [29].

#### 2.7.2. Histopathological Evaluation

The excision wound model was used for histopathological evaluation. A cross-section of full-thickness skin (wound tissue) specimen from each group (*n* = 3) was taken on days 5 and 15 of the experiment for histopathological evaluation. Samples fixed in 10% buffered formalin were dehydrated with a series of graded alcohols, clarified in xylene and embedded in paraffin blocks. 5 *μ*m thick sample sections were cut with a microtome (KD-2268 Manual Microtome) and stained with hematoxylin-eosin (HE) (Fisher Scientific). Stained sections were then examined under a Leica DME microscope (Leica Microsystems, Germany) (×20 objective).

### 2.8. Statistical Analysis

Data are represented as mean ± standard deviation (SD) or standard error of the mean (SEM) of at least three independent experiments. Statistical analyses were performed with the GraphPad Prism v6 software (GraphPad Software, San Diego, CA, USA). Continuous data were compared using a one-way, followed by Tukey's multiple comparison test after confirming a normal distribution and variance homogeneity. All *p* values are two-tailed, and *p* values less than 0.05 were considered significant (^*∗*^*p* < 0.05, ^*∗∗*^*p* < 0.01, and ^*∗∗∗*^*p* < 0.001).

### 2.9. Ethical Approval

BALB/*c* were housed according to the Council of the European Communities (86/609/EEC; November 24^th^ 1986) Directive regulating the welfare of experimental animals, and the experiments are approved by the Life Sciences and Health Research Ethics Committee (cer-svs) of the Institute of Biotechnology (University of Monastir, Tunisia; ethical approval no. 2019/02/I/CER-SVS/ISBM; 9 January, 2019).

## 3. Results and Discussion

### 3.1. Characterization of Collagen

**Figure 2:**
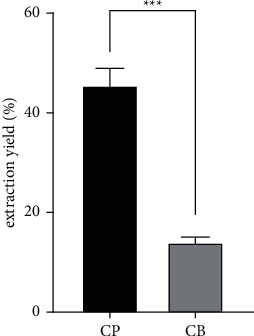
Extraction yield of marine and bovine collagen. CP: fish collagen; BC: bovine collagen. The values given represent the means (± standard deviation of the mean (SEM)) of three experiments (*n* = 3). The statistical significance of the results was assessed by the *t*-Student test. ^∗∗∗^*p* < 0,001 (significant differences from bovine collagen).

Acid-soluble collagen was extracted from two origins: marine (*Scyliorhinus canicula* skin) and bovine (hide sections from local cattle breeds). The yield of each extract was then determined. The yield extraction of marine collagen is 3 times higher than bovine collagen (Figure 2). The yield of bovine collagen is around 14.25%; however, the yield extraction of fish skin is around 48.66%.

Several reported similar results for marine collagen extracted from different species (*Lateolabrax japonicus*) (51.4%), mackerel (*Scomber scombrus*) (49.8%), horned sleeper shark (*Heterodontus francisci*) (50.1%) [30], and Nile perch (*Lates niloticus*) (58.70%) [31]. Other authors, Noitup et al. [32] and Nagai and Suzuki [33] reported a collagen yield extraction from *Oreochromis niloticus* of 38.84% and 39.4%, respectively. The main sources of industrial collagens are calfskin and bone. Nevertheless, these represent a high risk of bovine spongiform encephalopathy or transmissible spongiform encephalopathy [34]. Marine collagen thus provides an alternative to people who have issues about using other sources of collagen. Besides, due to its higher yield and extraction, fish collagen is gaining more interest for industrial purposes.

#### 3.1.1. SDS-PAGE Patterns of Acid-Soluble Collagen from Marine and Bovine Collagen

**Figure 3:**
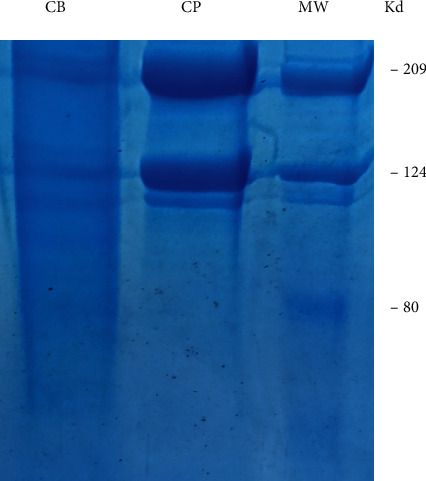
7% SDS-PAGE showing acid-soluble collagen from different origins. MW: molecular weight standards. CM: marine collagen; CB: bovine collagen.

SDS-PAGE pattern of collagen solutions was carried out. Our gels (Figure 3) showed identical profiles for both extracted collagens with a difference in band intensity. This is may be explained by the degree of purity of our extracts. SDS-PAGE patterns show three major bands of 209, 124, and 115 kD, which would correspond to the *β*, *α*1, and *α*2 chains, respectively. The profiles show hence a heterotrimeric structure with two *α* chains and one *β* chain. This configuration is characteristic of collagen type I [35].

In accordance with our study, several authors [36–38] previously reported that acid-soluble collagen showed properties of type I collagen, consisting of two *α*1 and one *α*2 chains. Indeed, the type I collagen molecule is a long triple helix formed by two *α*1 (I) and one *α*2 (I) chains. It belongs to the fibril-forming also called fibrillar collagen family and is present in connective tissues, mainly in the dermis, bone, tendons, ligaments, and cornea. Two genes, COL1A1 and COL1A2, encode *α*1 and *α*2 respectively [39]. In other types of collagen, the three helices are distinct, as, for example, in collagen IX, or identical as in collagen III.

Several collagen patterns similar to our work have been reported in fish skins: *Schyliorhinus canicula* [40], tiger globefish (*Takifugu rubripes*) [41], black drum (*Pogonias chromis*), sheep roundel (*Archosargus probatocephalus*) [42], Nile perch (*Lates niloticus*) [43], globe or fugu fish (*Lagocephalus gloveri*) [44], bamboo shark (*Chiloscyllium punctatum*) [45], and catfish (*Pangasianodon hypophthalmus*) [38] with two *α* chains (*α*1 and *α*2) and a *β* chain in their structure.

#### 3.1.2. Fourier Transform Infrared (FTIR) Spectra

**Figure 4:**
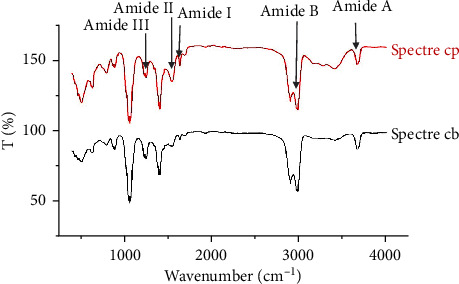
Fourier transform infrared spectra (FTIR) of marine collagen (CP) and bovine collagen (CB).

Results of FTIR spectra are shown in Figure 4. The two types of collagen exhibited comparable IR absorption as revealed by the pick peaking performed on the 4000–400 cm^−1^ interval. Figure 4 shows five major peaks absorption characteristics of the collagen molecule at the wave numbers of 3650 cm^−1^, 2980 cm^−1^, 1650 cm^−1^, 1550 cm^−1^, and 1250 cm^−1^, corresponding to amide A, amide B, amide I, amide II, and amide III, respectively, which are associated with the special triple helix conformation of intact fibrillar collagen [46–48]. Each peak in the FTIR spectra corresponds to the vibration of the functional groups of the molecules [49]. The amide A commonly associated with N-H elongation vibrations typical of intermolecular hydrogen bonding and is observed in the wave number ranging from 3400 to 3500 cm^−1^ [50]. The result recorded in the current study is higher than this range, suggesting that NH groups of this collagen were involved in hydrogen bonding, probably with a carbonyl group of the peptide chain [51]. In contrast, amide B corresponds to the asymmetric elongation vibration of CH2 bonds [52] and is found between 3080 and 2889 cm^−1^. Indeed, amide I represents the elongation vibration of C=O carbonyl groups along the polypeptide backbone with a characteristic wave number in the range of 1600 to 1700 cm^−1^ [53], and allows the formation of the secondary structure of polypeptides [54]. Amide II is associated with the N-H stretching bond coupled with the elongation vibration of C-N bonds [55] and typically occurs at 1550–1600 cm^−1^. Amide III represents a combination of C-N bond elongation vibration and N-H stretching bonds [56] and is observed in the wave range of 1220–1320 cm^−1^ [57].

Furthermore, the ratio of amide III to the peak at 1450 cm^−1^ is 0, 86 close to 1. This confirms that our collagens were not denatured during extraction and had a triple helix structure [58]. Absorption peaks around 1451–1450 cm^−1^ were also found. This corresponded considerably to the pyrrolidine ring vibration of proline and hydroxyproline. The band in the spectrum between 1200 and 1300 cm^−1^ is a unique “fingerprint” of the molecular conformation of collagen attributed to a particular tripeptide (Gly-Pro-Hyp) n. The peak at 2900 cm^−1^ which may be attributed to the C-H stretching vibration. Nevertheless, it is possible to notice differences in the width and height of the corresponding signals in several plots, which may be due to the different experimental conditions in each study (such as different solvents with different concentrations) [58]. Absorption in the peaks between 1000 and 1100 cm^−1^ is attributed to the C-O vibration due to carbohydrates [59].

In sum, the objective of this work was to extract and characterize acid-soluble collagen from fish and bovine skin. SDS-PAGE and FTIR confirmed that acid-soluble collagens were mainly composed of type I collagen with slight molecular structure differences. Both collagens showed properties of type I collagen consisting of two *α* chains and one *β* chain.

### 3.2. Fabrication of 3D Collagen Sponges Incorporated with Plant Extracts

**Figure 5:**
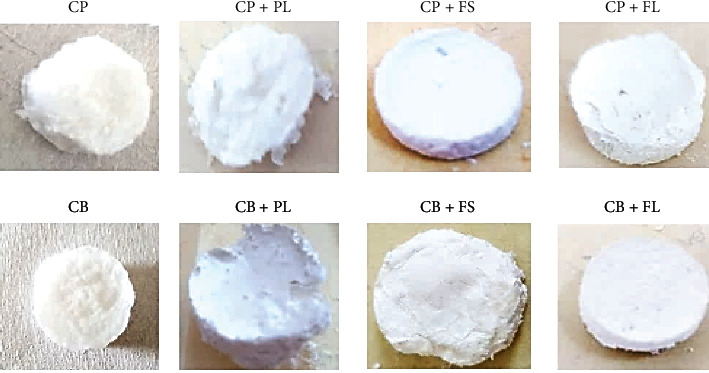
Macroscopic aspects of sponges. PL: *P. lentiscus* leaf extract; FL: *C. officinalis* leaf extract; FS: *C. officinalis* flower extract; CP: fish collagen; CB: bovine collagen.

In the current work, a collagen sponge soaked with bioactive extract is supposed to have an early synthesis of collagen and could exhibit significant reepithelialization in animals. Consequently, we designed collagen-based sponges to apply them as a temporary wound dressing. The macroscopic aspect of collagen varied with its source, marine and bovine collagen, do not have the same visual appearance. Bovine collagen proved to be much more difficult to solubilize than marine collagen. To achieve the same result, the latter had to be stirred in an acid solution for a further two hours (Figure 5).

### 3.3. *In Vitro* Biocompatibility of Collagen and Hydroethanol Extracts of *P. lentiscus* and *C. officinalis*

The first concern is about the conception of a novel dermal substitute without any biocompatibility issues. To determine whether collagen or plant extracts exerted a cytotoxic effect, we first exposed skin cells to increasing amounts of tested compounds (0 to 100 *μ*g/mL) for 24, 48, and 72 h prior to cell viability assays. After incubation of 3T3-L1 and HaCaT, cells for 24 h, 48 h, and 72 h at 37°C in an atmosphere enriched with 5% CO_2_, in the presence of different concentrations of compounds, the percentage of cell viability was calculated and compared to untreated cells.

**Figure 6:**
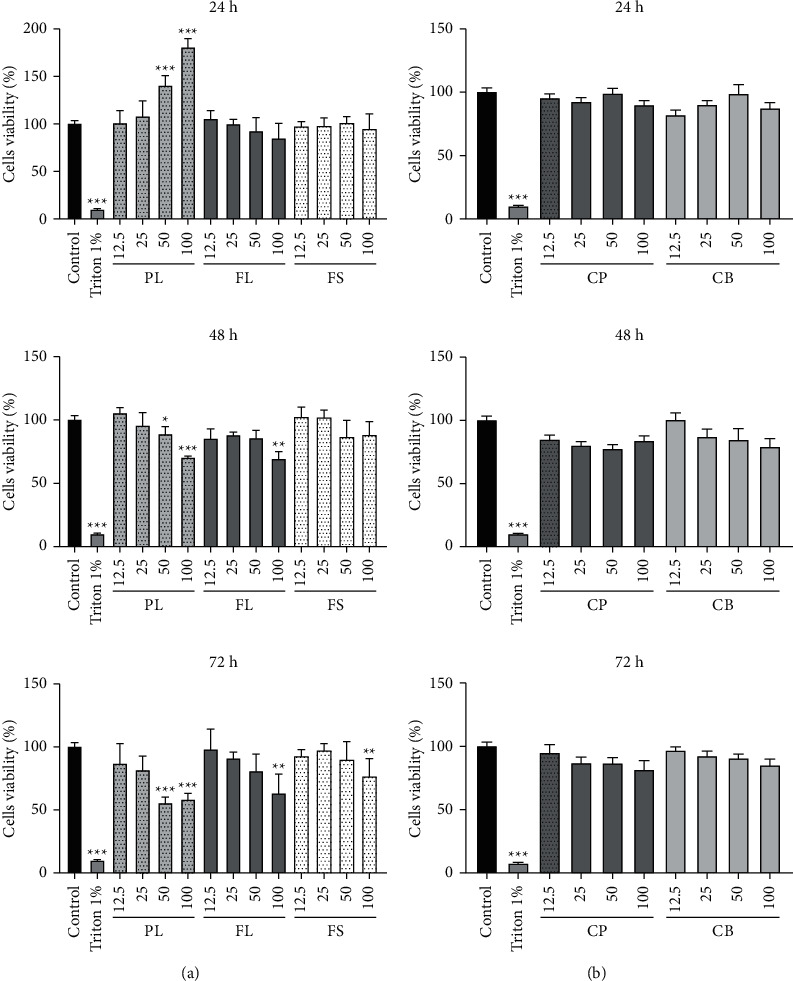
Effect of hydroethanol extracts (PL, FL, and FS) and various collagens (CP and CB) on the viability of keratinocytes (HacaT) after 24 h, 48 h, and 72 h incubation. PL: *P. lentiscus* leaf extract; FL: *C. officinalis* leaf extract; FS: *C. officinalis* flower extract; CP: fish collagen; CB: bovine collagen. HaCaT cells are treated with the different concentrations of collagens and ethanolic extracts (12.5 and 25 *μ*g/mL) for 72 hours of incubation, and the cell viability is assessed by the MTT assay. The values presented are means ± standard deviation and are representative of three independent experiments.

**Figure 7:**
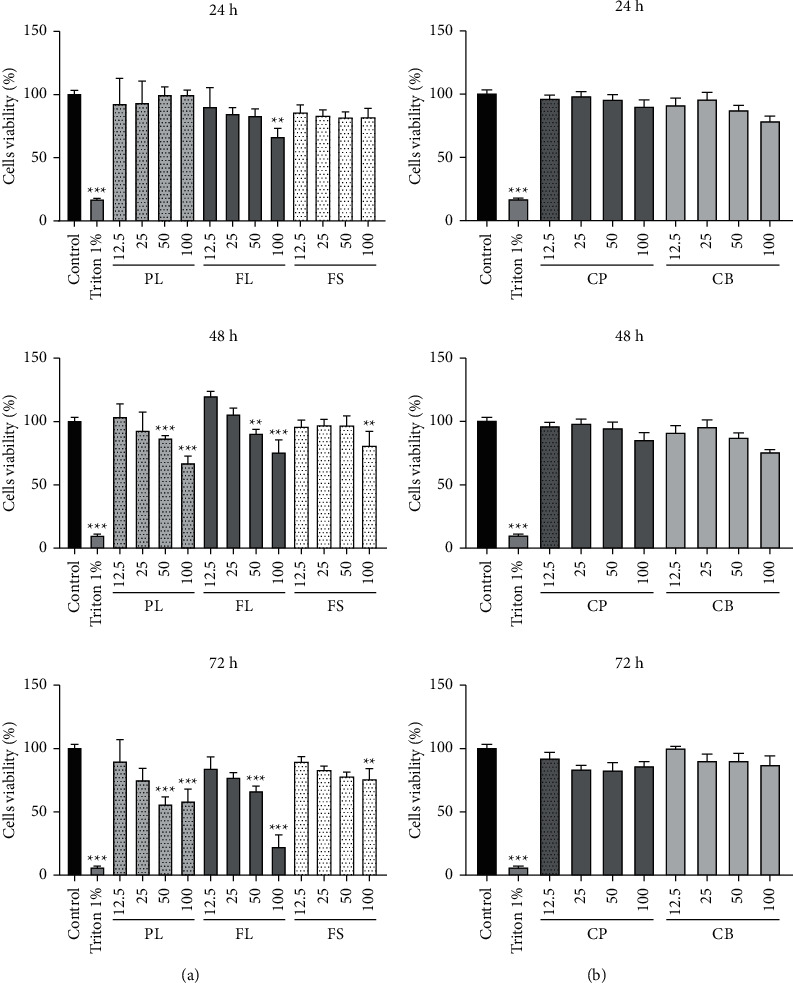
Effect of hydroethanol extracts (PL, FL, and FS) and various collagens (CP and CB) on the viability of fibroblasts (3T3-L1) after 24 h, 48 h, and 72 h incubation. PL: *P. lentiscus* leaf extract; FL: *C. officinalis* leaf extract; FS: *C. officinalis* flower extract; CP: fish collagen; CB: bovine collagen. 3T3-L1 cells were treated with different concentrations of collagens and hydroethanol extracts (12.5 and 25 *μ*g/mL) for three successive times of incubation 24, 48, and 72 hours, and cell viability are assessed by MTT assay. Cell viability is assessed by the MTT test. The values presented are means ± standard deviation and are representative of three independent experiments.

The results of the MTT assay shown in Figures 6and 7 reveal no cytotoxic effect of different extracts (CP, CB, PL, FL, and FS) against 3T3-L1 and HaCaT cells after 24 h of incubation. However, after 48 h and 72 h, a relative decrease in viability rate was recorded in cells treated with 50 and 100 *μ*g/mL of plant extracts. In whatever way, the lower doses of plant extracts (6.25–25 *μ*g/mL) induce a viability rate above 75%. However, cells exposed to increasing concentrations of collagen show a high survival rate at any incubation time.

Collagen is a biocompatible and bioactive polymer that, either alone or in combination with other materials, occupies a crucial position in the field of tissue engineering [60]. As revealed by these results of cytotoxicity, we confirm the *in vitro* biocompatibility of our extracts (plant and collagen) with fibroblasts and keratinocytes cells.

### 3.4. *In Vivo* Study

Both collagen and plant extracts possess fine biocompatibility with skin cells, fibroblasts, and keratinocytes. The use of collagen-based biomaterials has been extensively reported in the literature as a valuable alternative in wound healing and reconstructive surgery.

#### 3.4.1. Wound Closure Evaluation

**Figure 8:**
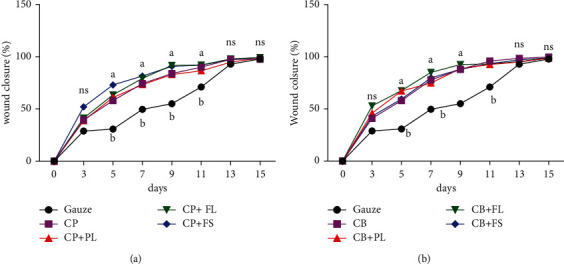
Evolution of the healing process of wounds covered with different sponges prepared from marine (a) and bovine (b) collagen in combination with plant extracts: PL,FL, and FS. Percentage wound contraction (*n* = 8) results are presented as mean ± SD. PL: *P. lentiscus* leaf extract; FL: *C. officinalis* leaf extract; FS: *C. officinalis* flower extract; CP: fish collagen; CB: bovine collagen. Different letters at the same sampling time denote significant differences (*p* < 0.05).

**Figure 9:**
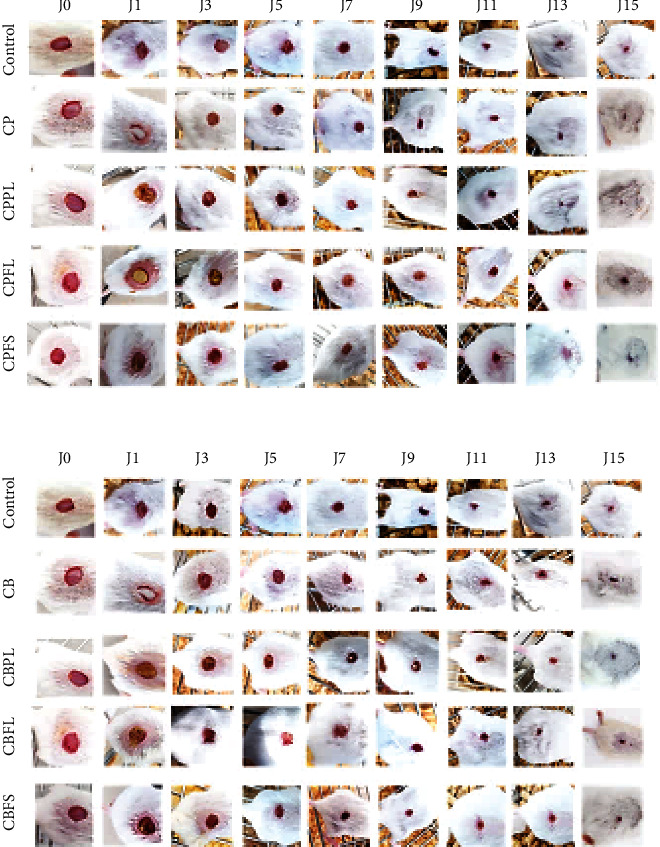
Photographic images showing the healing evolution of excisional wounds. PL: *P. lentiscus* leaf extract; FL: *C. officinalis* leaf extract; FS: *C. officinalis* flower extract; CP: fish collagen; CB: bovine collagen.

Figures 8 and 9 show the effect of the different sponges on wound closure. Results revealed that collagen sponge soaked with the bioactive extract significantly (*p* < 0.05) increased wound closure in treated mice. The period of healing was around 9 days for treated animals versus 13 days in the control group treated with conventional gauze. The mice treated with sponges prepared with marine and bovine collagen in association with extracts of *Pistacia lentiscus* showed the highest percentage of healing (50% of the wound was closed by the 3rd day of treatment). The groups treated with collagen plant extract scaffolds displayed a faster progress in wound closure from day 2 till day 7.

In general, we were able to show an improvement in healing due to the application of sponges.

The healing effect of *P. lentiscus* has also been tested on experimental burns [61]. Indeed, the wounds treated with the oil of lentisk showed a better evolution than those treated with Madecassol®, a commercial protective and healing cream.

Other authors have shown that *Calendula officinalis* extracts increase angiogenesis, epithelialisation, and metabolism of nucleoproteins, glycoproteins, and collagen leading to improved local circulation and granulation tissue formation [62]. *Calendula* flower extracts can also boost the healing of injured tissue and reduce pain [63]. This plant is used in traditional medicine to treat dermal pathologies [64].

#### 3.4.2. Histological Evaluation

**Figure 10:**
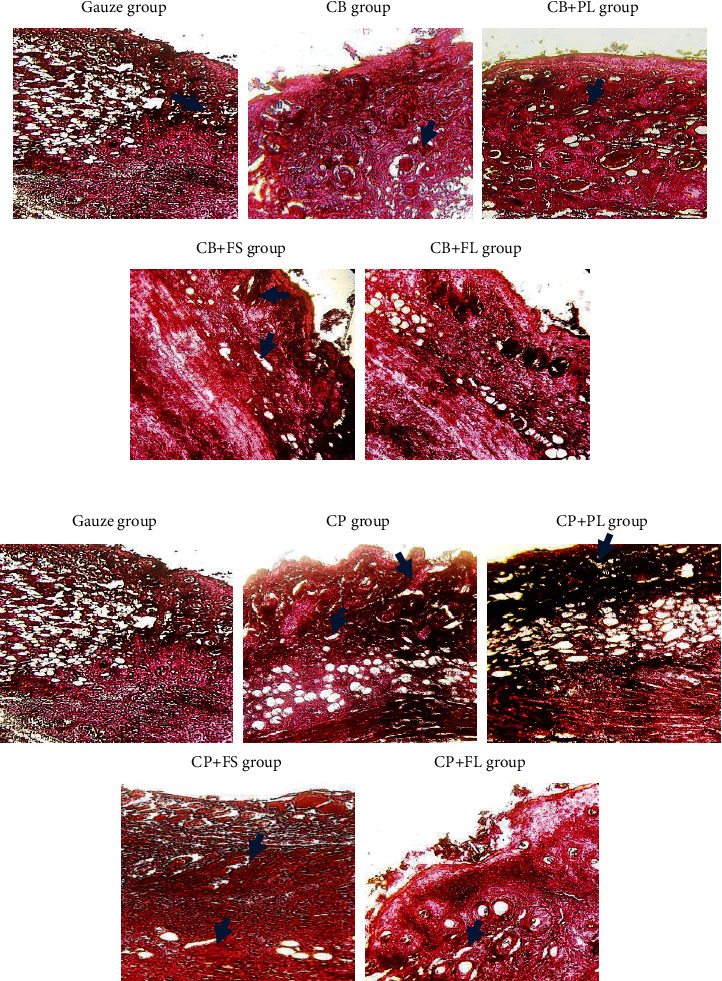
Light microscopy images of full-layer wounds (HE, X20) after 5 days. HE histological observation of wounds covered with vaseline gauze (VG) and current dressing CP, CP + PL, CP + FL, CP + FS, CB, CB + PL, CB + FS, CB + FL. Photomicrographs of histological sections stained in HE representative of the healing wound area in experimental BALB/*c* mice on day 5. The gas group (control) shows oedema and fibrinoleukocytic exudate and fibrose. The CP alone, CP + PL, CP + FS, CB + FL, and CB + FS groups show a cellular dermis, with fibroblast proliferation, depositing disorganized and misdirected collagen fibers. Capillary-sized blood vessels were visible (blue arrows). Scattered collections of inflammatory cells were observed in the CB only and CB + PL and CP + FL groups (×20).

**Figure 11:**
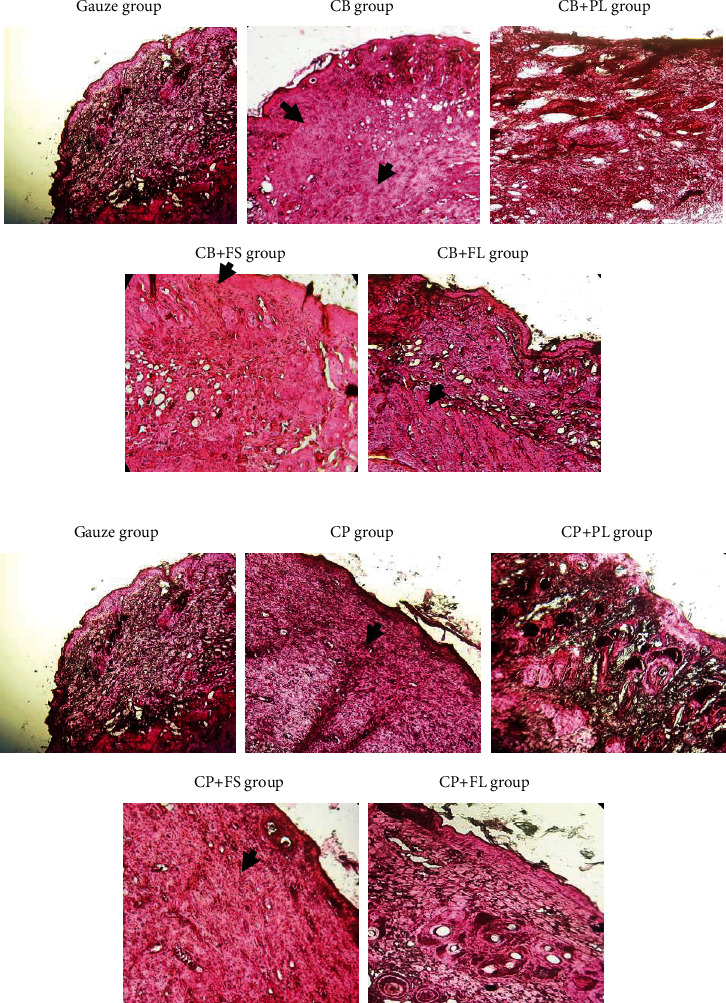
Photomicrographs of histological sections stained in HE representative of the healing wound area in experimental BALB/*c* mice on day 15. The gas group (control) shows oedema and fibrinoleukocytic exudate with the presence of numerous inflammatory and mononuclear elements (lymphocytes and plasma cells). Black arrow notes fibrosis. The CP + PL and CP + FL groups show immature, vascular, and oedematous granulation tissue (20×), while CB + PL and CB + FL show a greater content of proliferative spindle cells (20×). For the other groups (CP alone, CP + FS group, CB alone, and CB + FS), the granulation tissue was less vascular and more cellular (20×).

Histological evaluation was assessed on day 5 and day 15, for the treated and untreated groups. A comparison between the gauze group and treated animals is shown in Figures 10 and 11. The best results were obtained with animals covered by the prepared scaffolds when compared to the control.

As a result of 5 days of treatment with CP-plant dressings, there is full-thickness re-epithelialization, the *epidermis* is thin and well-organized, comparable to adjacent normal skin, and does not participate in the wound formation and healing process. The granular layer was well-formed and one-cell thick. The keratin layer is thin and made up of other keratin. The underlying dermis is lined with low cellular collagen, the fibers are horizontally oriented, and there is no inflammation or marked vascularity. Animals treated with a bovine sponge soaked with plant extracts showed comparable results when compared to each other and when the different groups were compared to the control.

On day 15, the inflammatory process still persists, but in a subacute mode with the presence of a moderate fibrinoleukocyte exudate. The signs of inflammation become even more rare with the focal presence of some inflammatory mononuclear lymphocytes within the fibrosis. However, mice treated with conventional gauze showed after 5 days of excision, oedema and a very large fibrinous leukocyte exudate and numerous inflammatory elements. The fibrosis is not very dense on day 5 and even on day 15. According to Shanmugam et al. [65], collagen scaffolds are able to regenerate the dermis and *epidermis* effectively and decrease infection at the wound site. Histology from sheep and human studies demonstrated that lyophilized, collagen implants were able to reconstitute after 5 weeks of the rotator cuff repair. Additional collagen was seen again on the implants, there was no trace of the implant in the rotator cuff tissue, and the new tissue was regenerated in its place [66, 67].

## 4. Conclusion

The application of 3D collagen sponge scaffolds soaked with *P. lentiscus* or *C. officinalis* leads to a significant increase in wound healing processes. The tested dressing was found to be effective in the functional recovery of the healing of wounds and histopathological changes. The tested materials showed a low cytotoxic effect against keratinocytes and fibroblasts and as infections are a major cause of morbidity in wound patients, the plant extracts added with collagen may accelerate the reepithelialization of patients and prevent infections. In this regard, the tested sponges are nominated as potential wound dressing materials. Further studies may cover complete physicochemical properties of the sponges such as SEM, contact angle, and ultimate tensile strength.

## Data Availability

The data used to support the findings of this study are included within the article.
